# Effects of Different Controlled Temperatures on Spanish-Style Fermentation Processes of Olives

**DOI:** 10.3390/foods10030666

**Published:** 2021-03-20

**Authors:** Daniel Martín-Vertedor, Thaís Schaide, Emanuele Boselli, Manuel Martínez, Rocío Arias-Calderón, Francisco Pérez-Nevado

**Affiliations:** 1 Technological Institute of Food and Agriculture (CICYTEX-INTAEX), Junta of Extremadura, Avda. Adolfo Suárez, s/n, 06007 Badajoz, Spain; 2 Nutrition and Bromatology Area, Department of Animal Production and Food Science, University of Extremadura, Ctra. de Cáceres, s/n, 06071 Badajoz, Spain; tschaide@alumnos.unex.es (T.S.); fpen@unex.es (F.P.-N.); 3 Faculty of Science and Technology, Free University of Bozen-Bolzano, Piazza Università 1, 39100 Bozen-Bolzano, Italy; emanuele.boselli@unibz.it; 4 Department of Engineering of the Agricultural and Forestry Environment, University of Extremadura, Avda. de Elvas, s/n, 06006 Badajoz, Spain; mmcano@unex.es; 5 National Institute of Agrarian and Veterinary Research (INIAV), Estrada de Gil Vaz, Apartado 6, 7351-901 Elvas, Portugal; rocioariascalderon@gmail.com

**Keywords:** Manzanilla Cacereña, Manzanilla de Sevilla, sensory analysis, thermal treatment, table olives

## Abstract

This work aimed to determine the effect of applying different temperatures during the fermentation process of Spanish-style table olives. ‘Manzanilla de Sevilla’ (southwest of Spain, Badajoz) and ‘Manzanilla Cacereña’ (northwest of Spain, Caceres) olives were processed at an industrial scale in table olive fermenters whose brine was subjected to different thermal treatments. One of the three conducted experiments found that maintaining brine at 20–24 °C over a 3-month period led to optimum firmness, better color indices, and greater free acidity and lactic acid bacteria populations in comparison to an unheated control. Furthermore, raising the temperature of the fermenter to 20–24 °C accelerated the fermentation process, provoking better lactic bacteria and yeast growth without affecting olive firmness. The higher fermentation rate (shorter time to completion) associated with temperature-controlled olives also reduced the marketing time of the final product. Controlling brine temperature led to a better aspect and color, higher acidity, lower bitterness, and better overall assessment of processed olives. In addition, ‘Manzanilla de Sevilla’ olives presented a higher phenolic content than ‘Manzanilla Cacereña’ olives. Preliminary evidence is presented suggesting that ‘Manzanilla Cacereña’ olives appear highly amenable to Sevillian-style processing. The present innovative work demonstrates the importance of applying different thermal treatments to brine to control the temperature during the industrial fermentation of table olives during the cold season.

## 1. Introduction

Mediterranean countries are increasing their efforts to produce table olives and olive oil [1]. To be more competitive in the global market, many Spanish companies need to reduce industrial costs and bring them in line with countries with much lower labor costs, such as Morocco, Tunisia, Argentina, Egypt, and Peru. Thus, to be more competitive in international markets, many Spanish businesses are studying the effects of small changes in the manufacturing process on final product quality. 

Spain is the greatest producer of table olives in the world [1]. The main table olive variety to be industrially processed in Spain is ‘Hojiblanca’, which is produced in Andalusia (southern Spain), followed by ‘Manzanilla de Sevilla’ and ‘Manzanilla Cacereña’. Table olive production is an important sector in Extremadura (southwest Spain), which registers 20% of national domestic production. ‘Manzanilla Cacereña’, cultivated in large areas of Caceres (northern Extremadura), is used to produce both high-quality table olives and extra virgin olive oil. Nowadays, ‘Manzanilla Cacereña’ is mainly processed for olive oil extraction and to obtain California-style black table olives in the northern region of Extremadura [2]. The final product of this variety has specific features, such as a fine pulp, low pulp/stone ratio, and good texture. At a national level, the second most representative olive variety is ‘Manzanilla de Sevilla’, which is one of the most important olive varieties from the region of “Tierra de Barros” (central region of the province of Badajoz). This variety is mainly used in the region to produce table olives [3,4]. 

Different classes of natural phenolics are present in both table olives and virgin olive oil [5]. These compounds have important health benefits in that they may protect against coronary heart diseases [6] and may prevent some types of cancer [7]. To the best of our knowledge, the phenolic profile of Manzanilla Cacereña and Manzanilla de Sevilla table olive varieties from Extremadura has yet to be determined in either fresh olives or fermented product [8].

Many research groups have based their studies on the selection of more appropriate microbial starters for olive fermentation [9,10]. Lactic acid bacteria positively contribute to the fermentation process, while some yeasts have also been seen to positively impact the quality characteristics of the final product [11,12,13]. 

Other experimental variables, such as temperature or salt concentration, have been studied throughout the fermentation process with the goal of obtaining better final products than those produced through conventional elaboration [6,14,15,16]. In addition, since the olive harvest in Extremadura begins in September, the fermentation process may suffer from winter frost in December. At low temperatures, the fermentation process is slower and can even get stuck during the coldest months. Fermentation may restart later during the springtime due to rising temperatures. For this reason, the entire fermentation process can last as long as the whole wintertime plus the beginning of spring. However, during this long period, abnormal and uncontrolled fermentation may take place. These lead to off-flavors and lower final product quality. Moreover, the business will suffer from an excessively short commercial period. 

Important quality improvements may be achieved through temperature control strategies. Temperature sensors and equipment that apply different heat treatments may be easily installed, even by small companies. A thermostat-controlled temperature of around 21 °C has been recommended for correct table olive fermentation [16]. However, such equipment is not commonly used by olive processing companies because of its high energy demands. Thus, a detailed analysis must be performed by the company, which compares the benefits of increasing brine temperature above regular winter temperatures with the additional costs.

To the best of our knowledge, this is the first time that the characteristics of fermenters employed with two olive varieties from different geographical areas with different climatic and environmental conditions have been studied. It is also the first time that such an examination has been made of Spanish-style olive fermentation at an industrial scale. Different artificial thermal treatments were used to heat the brine surrounding olives during the fermentation processes. The final product quality was evaluated to determine the effects of different temperature control methods and optimize the industrial fermentation process. To meet this aim, physical-chemical (color, firmness, pH, and free acidity) and microbiological parameters (viable counts) were evaluated during the elaboration process, while the HPLC phenolic fingerprint and sensory profile were also determined in the final product.

## 2. Materials and Methods

### 2.1. Experimental Design

Olive fruits grown in good phytosanitary conditions were harvested by hand at the green stage of maturation. They were then subjected to three different processing experiments according to Spanish-style elaboration processes described by Schaide et al. [12]. For all three experiments, a completely randomized design with a factorial arrangement was employed. In all cases, spontaneous fermentation occurred between October and March 2018 (160 days). 

The three types of industrial-scale temperature-controlled experiments were as follows: 

Experiment 1. This experiment took place in the northwest of Spain (Caceres) and used Manzanilla Cacereña variety olives. During the fermentation process, three commercial fermenters were kept at normal room temperature (to be considered as process controls), and another three fermenters were located in a warehouse where heat storage capacity was improved via more efficient heat retention (due to it being sheltered). The fermenters were made of polyester reinforced with fiberglass. Both systems used 16,000 L capacity filters filled with 10,000 kg of table olives.

Experiment 2. The second experiment was performed in Badajoz. Three fermenters for each selected temperature treatment were studied during the fermentation process of Manzanilla de Sevilla olives. These three fermenters artificially controlled brine temperature throughout fermentation so that it stayed at 20, 22, and 24 °C, respectively. Three temperature control fermenters containing olives whose brine temperature was not modified (control) were also used. The fermenters were made of polyester reinforced with fiberglass. Heat was provided through a system that circulated hot water, whose heat transfer to the brine was monitored by means of a temperature sensor. Fermenters had a capacity of 236 L and were filled with 190 kg of table olives.

Experiment 3. The third experiment was carried out at a Spanish company (Badajoz) with Manzanilla de Sevilla olives. Eighteen tanks were used. Specifically, six old fermenters with little heat control capacity (control) (Old chambers), six fermenters at normal room temperature located under the floor (buried), and six fermenters constructed out of a new, more winter-resistant material (isolation). The fermenters were made of polyester reinforced with fiberglass, aside from those with high isolation chambers, which were sprayed with projected polyurethane. Furthermore, the buried fermenters and isolation chambers were heated by exposing them to air, which was artificially heated to 25 °C, under the floor. Fermenters had a capacity of 16,000 L and were filled with 10,000 kg of table olives.

**Figure 1:**
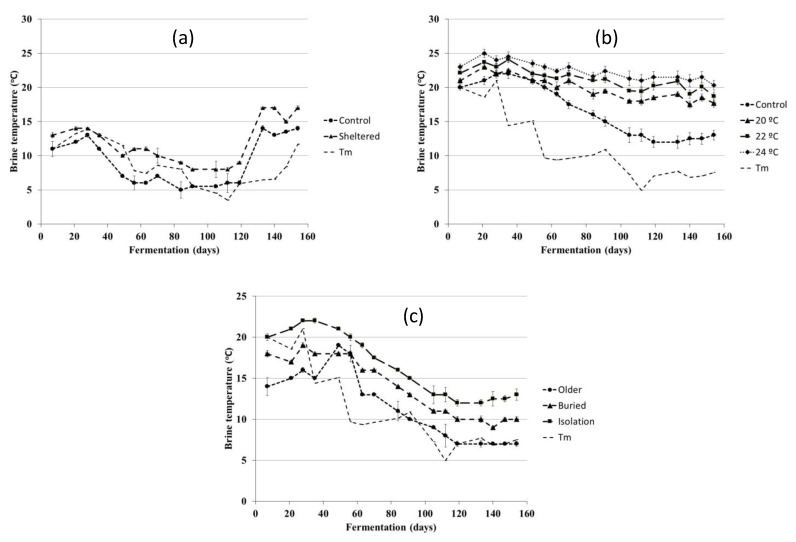
Brine temperature measured in each of the tanks during the fermentation process. Each figure also presents average ambient temperature (Tm) as recorded by the closest weather station. Experiment 1 (**a**), experiment 2 (**b**), experiment (**c**).

For all experiments, the brine temperature was measured weekly in each of the tanks during the fermentation process (Figure 1). A PT-100 digital calibration probe with a 2-m steel sheath was used to make this measure. Brine temperature was measured at three different depths inside each tank, and average values were calculated. Average ambient temperature (Tm) was also noted from the nearest weather station to the companies under study. 

Color, firmness, pH, and free acidity of table olives were measured throughout the whole fermentation process. Samples were collected at different stages of fermentation (30, 40, 45, 60, 95, 120, and 160 days from fermentation start), and the phenolic profile of the final product was characterized following fermentation. 

### 2.2. Physical–Chemical Parameters

Maximum resistance to penetration was determined by means of a Texture Analyzer TA-XT2 (Stable Micro Systems, Surrey, UK) [12]. The texture test was carried out on fifteen olive fruits for each sample, penetrating 2 mm into the center of the olive. Olive color was measured according to the color index proposed by Cabrera-Bañegil et al. [16] using a UV-Vis-2450 spectrophotometer (Shimadzu, Tokyo, Japan). The pH was evaluated using a pH meter (Crisol, Model Basic20) [12]. To determine free acidity, 10 mL of brine was titrated with NaOH (0.2 mol/L) in the presence of phenolphthalein. Outcomes were reported as % (*w/v*) of lactic acid [12]. 

### 2.3. Phenolic Extraction and HPLC Analysis of the Phenolic Profile of Table Olives

Phenolic extraction and characterization of the phenolic profile were performed according to the methodology described by Cabrera-Bañegil et al. [17]. This was performed via chromatographic separation. An Agilent 1100 series HPLC system (Hewlett–Packard, Waldbronn, Germany) equipped with a diode array detector (DAD) and fluorescence detector (FLD) was used.

### 2.4. Microbiological Analysis 

Olive and brine samples (10 g) were extracted throughout the fermentation process. They were mixed with 90 mL of sterilized peptone water solution and were homogenized with a Stomacher 400 circulator (Seward Inc., London, England) at 300 rpm for 3 min. Decimal dilutions of brine were prepared with sterile 0.1% (*w/v*) peptone solution. These dilutions were injected onto the surface of different solid growth matter to examine the accumulation of common microorganisms during fermentation. Lactic acid bacteria, viable mesophilic microorganisms, yeasts, and molds were analyzed, as were pathogens and microorganism indicators, such as enterobacteria, coliforms, *Pseudomonas* and *Bacillus cereus* [12]. Lactic acid bacteria (LAB) were quantified using Man-Rogosa-Sharpe (MRS) Agar. Culture plates were incubated under micro-aerophilic conditions (AnaerocultC mini, Merk, Darmstadt, Germany) at 30 °C for 72 h. Viable mesophilic counts were estimated on a plate count agar (PCA) incubated at 30 °C for 72 h. The quantity of different yeasts and mold was estimated according to a yeast extract glucose chloramphenicol (YGC) agar medium (Merck, Darmstadt, Germany). Colonies were counted following incubation at 25 °C for 5 days. Microbial counts of olives and brine samples were determined in triplicate for each treatment. Outcomes were expressed as log_10_ cfu·g^−1^ of olive and brine with a detection limit of 10 cfu·g^−1^.

### 2.5. Sensory Analysis

Sensory assessment of table olive samples was conducted at the end of the fermentation process by a panel of 12 expert tasters belonging to a multidisciplinary team from the CICYTEX (Technological Institute of Food and Agriculture) research center. For this, a scoreboard was prepared according to a standardized method previously developed by the authors [12]. The sensory properties of the brine and olive fruits, including aspect, hardness, acidity, saltiness, bitterness, taste, fibrousness, crunchiness, and defects (off-flavors), were evaluated by members of the expert panel, and a global assessment was made.

### 2.6. Statistical Analysis

One-way ANOVA and Duncan’s multiple range test were performed to determine significant differences between experimental treatments. SPSS 18.0 software (SPSS Inc., Chicago, IL, USA) was used to perform ANOVA (*p* < 0.05) analysis. Outcomes were expressed as mean values and corresponded to the thermal treatments applied in each experiment. Statistical significance was accepted at the level of *p* < 0.05. Data were expressed as mean ± standard deviation. 

## 3. Results and Discussion

### 3.1. Effect of Different Thermal Treatments on Temperature within the Fermenter 

The different materials making up the fermentation tanks and the different techniques used to heat the brine were studied to uncover differences in olive quality. For this reason, the temperature inside the tanks was measured weekly (Figure 1). As shown by the results, the application of thermal techniques in the fermenters made it possible to increase the temperature inside. 

Temperature differences were more notable when the temperature of the fermenter was artificially modified to reach different temperatures through the application of a heat exchanger (experiment 2). At the beginning of the fermentation period, small temperature differences began to be found with respect to the control treatments. Fermenters made out of better isolating materials were able to retain 2.47–2.85 °C more than fermenters made out of older materials. This is a good outcome as it highlights that the simple introduction of fermenters with better isolating properties can achieve higher temperatures which potentially results in better fermentation. 

While applying heat by placing a heat exchanger inside the fermenter also increases the inner temperature, this practice is more expensive. Thus, the additional human resources needed to carry out this work and energy cost should be assessed. In this case, benefits were obtained when the temperature of the brine was artificially modified, with the measured temperature of the fermenter rising from 3.28 (22) to 5.82 °C (24 °C) with respect to the control. Further, at the end of the fermentation period, the temperature had increased above control by 5.19 and 8.12 °C, to 22 and 24 °C, respectively.

We would like to highlight that notable outcomes were also produced in relation to fermenters made of polyester reinforced with fiberglass and sprayed with projected polyurethane when subjected to air heated artificially to 25 °C under the floor. Applying heat underneath the fermenter is a simple practice that requires little human resources and economic investment. In this case, the temperature rose 5.03 °C relative to the control treatment.

Finally, it should be noted that when internal fermenter temperatures were compared with average outside temperatures, a substantial increase in brine temperature was also observed. As can be seen in Figure 1, control treatment temperatures were slightly higher than the ambient temperature. However, it should be noted that these differences were much greater in relation to the other fermenters, especially at the end of the fermentation period.

### 3.2. Effects of Different Thermal Treatments on Microbiological Properties 

Table 1 presents the number of lactic acid bacteria and yeast detected in the three conducted experiments. Pathogenic bacteria (*Enterobacteriaceae*, coliforms, *Pseudomonas,* and *Bacillus cereus*) were not detected in any of the batches examined in the three experiments. This is a positive result since it suggests that the use of different fermenter materials to maintain the temperature does not lead to an increase in the growth of microorganisms, which are undesirable for the fermentation process. Hurtado et al. [18] found a non-*Pseudomonas* colony emerged in relation to a temperature increase from 17 to 24 °C.

Generally speaking, LAB and yeast growth was better in all three experiments in batches subjected to controlled temperatures than those in control fermenters (Table 1). During the fermentation period, different temperatures were reached inside the fermenters because different heat treatments were applied. Control fermenters had lower temperatures than controlled temperature fermenters whose temperature varied from 2.85 (experiment 1) to 5.82 °C (in a fermenter whose brine was set at 24 °C in experiment 2) and 5.03 °C (in more water-resistant fermenters, due to the presence of an isolation chamber, in experiment 3). This could have provoked the differences seen in the development of microorganisms. For instance, LAB did not grow well in these fermenters, which lead to a slow fermentation rate relative to that seen in controlled temperature fermenters. 

Initially, all batches presented low values of lactic bacteria and yeast. However, in the first 20 days, there was a large increase in the values of these microorganisms. The number of LAB and yeast stabilized at different moments depending on the experiment under consideration. Fermentation in experiment 2 was more rapid, with the maximum number of microorganisms being reached on day 20. In experiment 1, the number of LAB and yeasts stabilized on day 60. Finally, fermentation in experiment 3 was slower, stabilizing on day 100. These differences can be explained by the different methods used to control fermenter temperature, and the different temperatures reached in each experiment. In experiment 2, fermenter temperature was controlled artificially at 20, 22, and 24 °C. This favored the growth of microorganisms. In contrast, temperatures in experiment 1 were lower, although they increased over the last 40 days of fermentation. At the end of fermentation (day 160), the number of microorganisms had decreased in all samples. Total mesophiles and LAB were higher in the table olives produced using controlled temperature fermenters than those produced following control processes. 

As expected in all controlled temperature fermentations, the population of LAB increased higher than the yeast population during fermentation. Finally, a decrease in lactic bacteria was observed at the end of fermentation. This was probably due to a decrease in nutrients and the concentration of several toxic compounds in the fermentation medium. As a consequence, the final quantity of LAB and yeast was similar on day 160. However, this trend was reversed in control fermentations, with larger populations of yeast being found than lactic acid bacteria. 

Thus, the application of different measures to control internal fermenter temperatures was useful for maintaining higher temperatures throughout the overall winter period. Temperature control permitted optimal development of the LAB population and improved the quality of the final product. On the other hand, experiment 3 proved that simply using fermenters made out of better insulating material, which protected them from high outside temperatures, caused better microbial development. Moreover, in experiment 2, the use of a heat exchanger improved the fermentation process. This was easy to implement; however, the use of this technique implies additional energy expenditure, which should be assessed by the industry.

In the present study, fermentation largely occurred through LAB, although a significant number of yeasts was present. In general, most of the fermenters, with the exception of the controlled temperature fermenters used in experiment 2, showed lower levels of LAB than those seen following other olive fermentations [11,19]. The application of high temperatures (up to 18 °C) seems to be important for enhancing lactic acid bacteria growth. This fact could be related, at least in part, to the diffusion of nutrients in the brine [20]. De Florio et al. [21] indicated that maintaining temperatures controlled at 25 °C provoked reduced growth during the fermentation period. In relation to yeast, the present study found similar outcomes during fermentation to those reported by other studies performed with olives subjected to Spanish-style processes [11,19]. Despite the fact that yeasts have, in some cases, been associated with alterations in olives, yeast has been proven to be of great importance to the quality of table olives [11,12].

### 3.3. Effects of Different Thermal Treatments on Physical–Chemical Properties 

**Figure 2:**
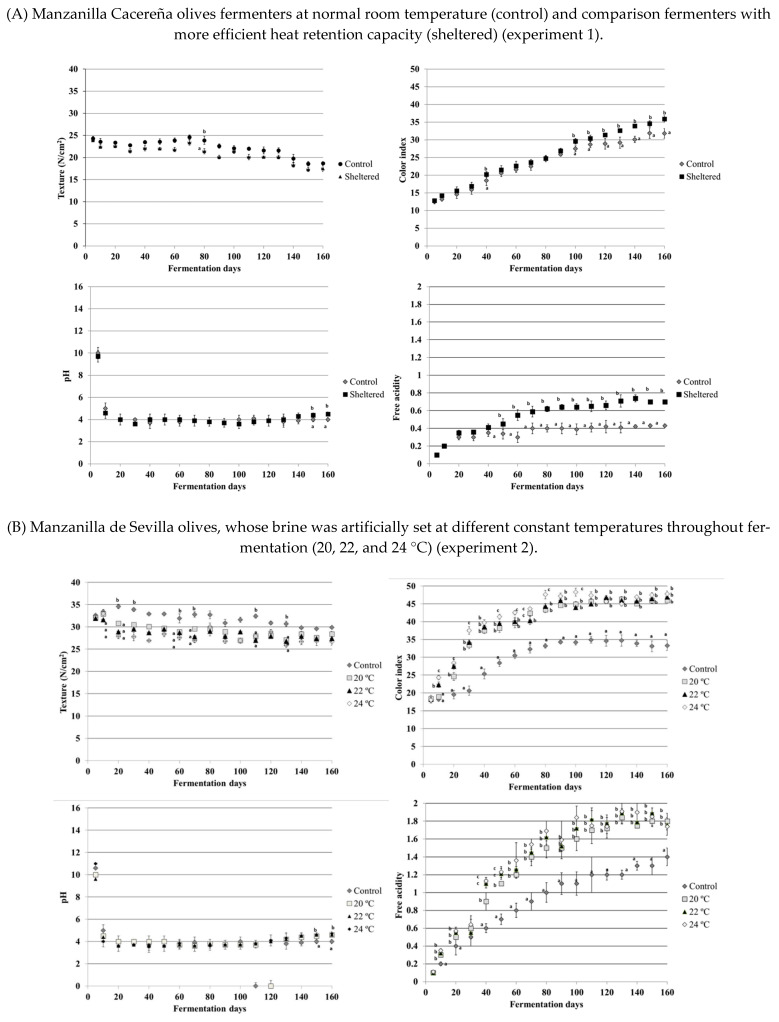

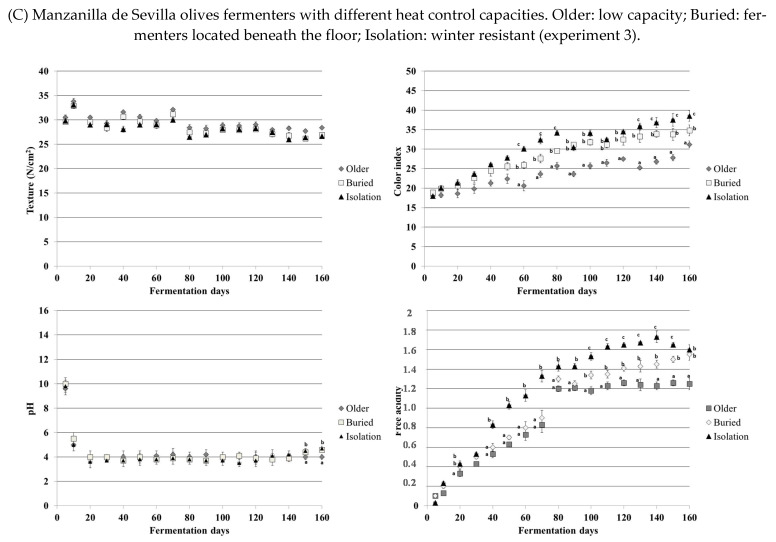
Physical–chemical fermentation parameters measured in the three experiments: texture (N/cm^2^), color index, pH, and free acidity (%). Different superscript letters within the same column indicate statistically significant differences (Duncan’s test, *p* < 0.05) between thermal treatments.

Physical–chemical fermentation parameters are shown in Figure 2. As can be seen in the figure, olive texture in the three treatments was constant up until day 80 of fermentation. Following day 80, texture decreased slightly and consistently until the end of fermentation. Control olives appeared to show a slight tendency towards greater texture, although differences were not significant. In fact, at the end of fermentation, both treatments presented similar values. The olive texture is an important aspect for the final consumer since soft olives provoke rejection by the consumer. In the present study, the softest olives were those produced in experiment 1. A priori, it was expected that this parameter would be modified with respect to the control treatment. However, olives were not softened during production when the temperature inside the fermenter was substantially modified. The temperature increases inside the fermenter accelerated the fermentation time. However, the olive firmness was not affected. There is a lack of scientific literature examining the effects of the fermenter temperature on the final olive quality. Cabrera-Bañegil et al. [16] showed that thermal brine treatments during the fermentation process did not affect firmness in Manzanilla Cacerena olives at three differential stages of maturation.

In addition, olives submitted to these heat treatments presented exponentially greater color indices (Figure 2). Data pertaining to the first days of both treatments were similar. However, color indices presented significant differences between treatments. Indeed, at the end of fermentation, the control treatment had a color index that was 4–5 points lower than that of the other fermenters. Temperature-controlled olives (experiment 2) presented higher color indices at the end of the fermentation process. Differences were greater when the brine was artificially heated. Heat produces conditions that are favorable to the development of microorganisms that facilitate fermentation (Table 1). After heating brine at different stages of olive fruit maturation, other research has found better final table olive color, with color being more quickly obtained via thermal application [16].

The pH of the brine decreased during the olive fermentation process. Slight differences were found between treatments at the end of the process, likely due to the end of fermentation (Figure 2). In addition, acidity also increased during olive fermentation. Increases were similar in both treatments up until day 40 of fermentation. Following day 40, higher values of acidity were observed in the treatment with higher heat conservation capacity and when the thermal treatment was applied until the end of fermentation. The maximum acidity observed in this treatment was 0.8%. In the control treatment, acidity values increased to almost 0.4%, and these values were maintained until the end of fermentation. Furthermore, olives produced in older fermenters presented the lowest acidity values, while the treatment applying artificial thermal conditions achieved the highest values of free acidity. Free acidity behavior during fermentation was observed to be similar in ‘Moroccan Picholine’, ‘Languedoc Picholine’, ‘Ascolana’, and ‘Sevillana’ varieties [22]. ‘Sevillana’ and ‘Negrinha de Freixo’ olives reached final maximum average values of 1.0% (*w/v*) and 0.9% (*w/v*), respectively [23]. The free acidity of brine increased progressively with increasing fermenter temperature. The growth and development of microorganisms promoted by the heat treatment speeded up the olive fermentation process. This phenomenon was influenced by the temperature surrounding the olives. Our results showed higher free acidity in the control treatment conducted in the warmest region (southwest of Spain) than the control treatment conducted in the coldest region. Similar results were obtained by Romero-Gil et al. [20], who indicated that higher brine solution temperatures clearly favored lactic acid fermentation. This is exactly what happened in the present study, as seen through the greater microbiological growth, which also shortened the time needed to elaborate the final product. This outcome was more pronounced in olives that underwent artificial temperature control, for example, by applying hot air or thermally treating the brine. Older fermenters presented the lowest acidity values due to the low brine temperatures, which slowed down microorganism development. Once again, olives were observed to ferment more quickly when they were stored in temperature-controlled fermenters. While this fermenter ensured an acceptable physical–chemical quality of the final olive by retaining more heat, the greater initial investment required should be considered. 

### 3.4. Effect of Thermal Treatments on the Phenolic Profile

The phenolic compounds found in the different table olive samples at the end of the fermentation process are reported in Table 2. Irrespective of the temperature applied, hydroxytyrosol was the main phenolic compound recorded in all experiments, followed by tyrosol and oleuropein. 

Some differences were found when comparing the control and temperature-controlled batches produced in the different experiments. In experiments 1 and 2, tyrosol concentrations were higher in control fermenters. Further, in experiment 1, the same outcome was found for hydroxytyrosol concentrations. With regards to the other compounds, differences were found between batches, although the highest concentration varied depending on the experiment and the type of fermenter used, regardless of whether temperatures were controlled or not. In this sense, in experiments 2 and 3, significant differences were found between the batches for one of the most important phenolic compounds, oleuropein. In experiment 2 alone, oleuropein concentration was higher in control batches. Further differences were also found between batches produced in experiments 1 and 2 in relation to apigenin, luteolin, verbascoside, vanillin, PB1 (procyanidin B1), PB2 (procyanidin B2), and epicatechin. The nature of these differences depended on the experiment and batch analyzed. 

In another sense, differences relating to phenolic compounds were found when comparing different olive varieties. The highest concentrations of hydroxytyrosol and tyrosol were found in experiments 2 and 3, which were carried out using Manzanilla de Sevilla olives as opposed to the Manzanilla Cacereña olives used in experiment 1. In contrast, oleuropein and other compounds showed similar concentrations in the three experiments. Moreover, most of the phenolic compounds, such as hydroxytyrosol, tyrosol, oleuropein, vanillin, and PB1, were significantly higher in Manzanilla de Sevilla table olives than in Manzanilla Cacereña table olives, while apigenin and luteolin content was higher in Manzanilla Cacereña olives. Flavan-3-ols, such as catechin, epicatechin, procyanidin B1 (PB1) and procyanidin B2 (PB2), and benzoic acids, such as vanillic acid, were present in lower concentrations than the aforementioned phenolic compounds in both varieties. Other minor phenolic compounds, such as apigenin, luteolin, and luteolin-7-*O*-glucoside, were quantified. 

Results from the present study point to a relationship between phenol concentration and olive variety. This is in accordance with other studies, such as that conducted by Kiai and Hafidi [22], who found similar hydroxytyrosol levels in Manzanilla de Sevilla fermented olives and Manzanilla de Sevilla olives treated with NaOH. Franco et al. [8] quantified phenolic compounds in unfermented olives and found higher concentrations in Manzanilla de Sevilla olives relative to Manzanilla Cacereña olives. In contrast, hydroxytyrosol and tyrosol concentrations were significantly lower in a study conducted by Lodolini et al. [24] on Spanish-style fermentations with ‘Ascolana Tenera’ olives. Pistarino et al. [25] found lower hydroxytyrosol concentrations in fermented ‘Taggiasca’ variety table olives, although similar tyrosol concentrations were reported.

In addition, fermentation temperatures in the present study did not seem to have a significant influence on final phenol concentrations, despite different microbial counts being found during fermentation. In contrast, previous studies conducted by the present research group using ‘Carrasqueña’ olive varieties found higher phenolic contents when brine was heated to 22 °C relative to control [16]. Further, Pistarino et al. [25] indicated that increasing fermentation temperatures from 23 to 37 °C generally reduced phenol content. These authors related this fact to greater *Lactobacillus plantarum* and *Saccharomyces cerevisiae* development during the fermentation process. 

These findings point to an association between the olive variety and the phenol profile. The phenolic profile could, therefore, be potentially used as a varietal marker. This characteristic is of great importance to olive mills as increasing competition between companies urges the need for suitable quality assurance measures and effective quality control by public authorities. Nonetheless, further studies should be conducted taking into consideration irrigation or fertilization conditions and growing conditions, such as the soil or climate, to verify the reliability of this hypothesis. 

### 3.5. Effect of Different Thermal Treatments on Sensory Properties

**Figure 3:**
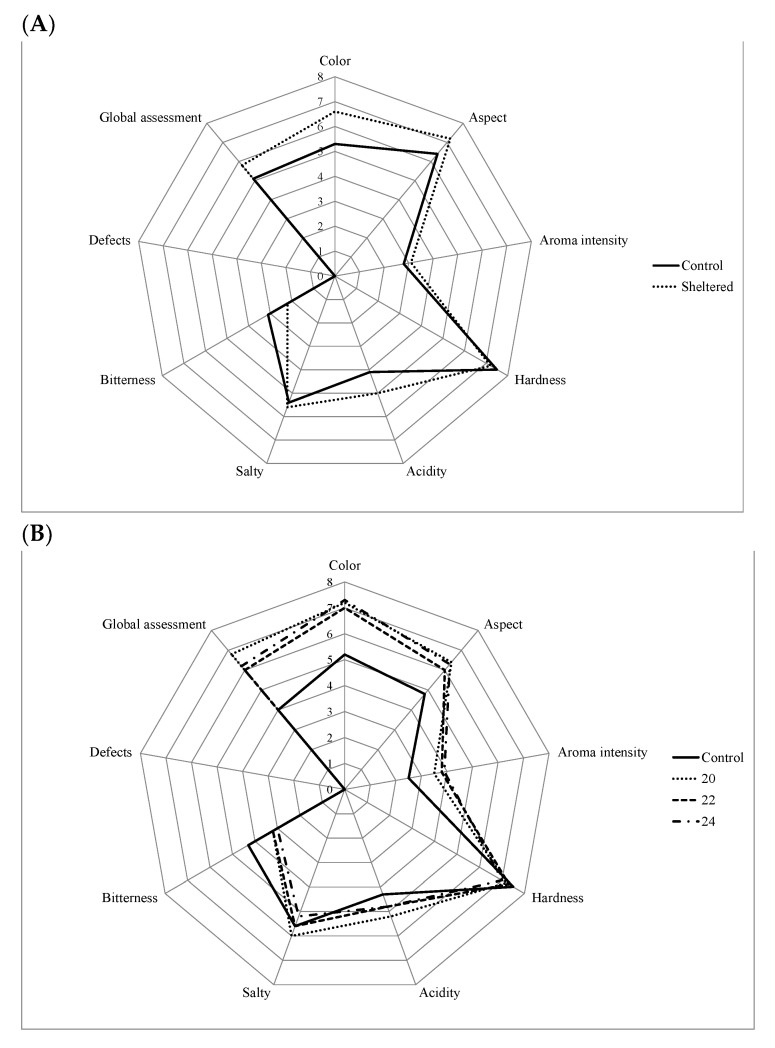

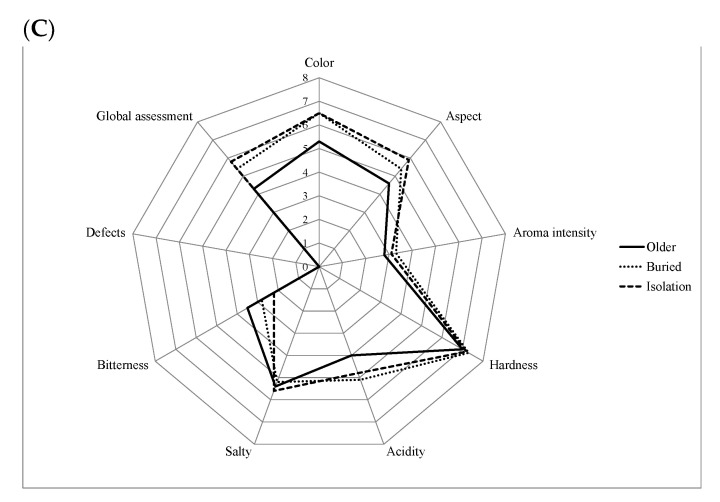
(**A**) Manzanilla Cacereña olive processed in control and sheltered fermenters (experiment 1); (**B**) Manzanilla de Sevilla submitted to different thermal treatments inside the fermenters and control treatment (experiment 2); (**C**) Manzanilla de Sevilla variety processed in older, buried, and isolated fermenters (experiment 3).

Sensory analysis was performed of the final table olives, and the results are shown in Figure 3. Widely varying sensory properties can be observed for all of the olives obtained from the different study experiments.

No significant differences were found (*p* < 0.05) in the color of evaluated controls in any of the three experiments. With regards to overall olive assessment, lower scores were attributed to samples fermented without temperature controls. Overall assessment scores differed by one point between varieties, with higher scores being reported for Manzanilla Cacereña than for Manzanilla de Sevilla in experiments 1, 2, and 3, respectively (Figure 3). In experiment 1, we can verify that tasters reported better color quality (Figure 3A) in olives that were fermented in tanks protected from the outside environment. Similarly, sensations of acidity were greater, and a better aspect was produced in olives fermented in sheltered fermenters. The most bitter table olives were obtained using non-temperature-controlled fermenters (control samples). This is to be expected since these fermenters had a lower microbial load (Table 1), as explained in the previous experiment, and, therefore, presented reduced oleuropein breakage. Since oleuropein is the main compound responsible for the bitter taste, this is an important attribute to highlight, despite the fact that some Spanish-style olives undergo lye treatment to reduce bitterness. Nevertheless, olives stored in fermenters with adapted structures were less bitter. Thus, it is necessary to emphasize that the use of fermenters with good isolation properties alone produces fairly interesting results.

Tasters in experiment 2 (Figure 3B) rated olives more positively in relation to color, appearance, acidity, and aroma parameters when olives had been fermented following forced thermal treatments than when they had undergone control conditions. However, olives were more bitter in control conditions. In experiment 1, greater microbiological content provoked a minor sensation of bitterness in olives subjected to brine temperature control. Thus, from the results obtained, we can observe that heating brine to determined temperatures could provide a strategy for accelerating the fermentation process. This would enable olives to be put on the market earlier as they present good physical, chemical, and sensory qualities. Sensory analysis outcomes pertaining to experiment 2 were in line with those presented in previous work characterizing ‘Aceituna Manzanilla’ and ‘Gordal Sevillana’ varieties [26].

In accordance with the experiment described above, fermented olives in experiment 3 presented better color, appearance, and acidity properties than those fermented in control conditions. Thus, the use of fermenters located beneath the floor and constructed out of better materials, which are more resistant to the cold season, is a good strategy for producing more highly rated olives. Furthermore, the use of artificial hot air, applied under the floor, also improves other sensory qualities of the olive, such as appearance or bitterness.

The bitter taste was one of the parameters for which significant differences were found (Figure 3). The bitterest table olives were obtained by using non-temperature-controlled fermenters (control samples). Despite the differences found, the bitter taste did not provoke rejection of the tasters trained for this study which is, in itself, a positive outcome.

Tasters rated average orthonasal aroma to be 3 points, with no significant differences found between experiments. Similarly, no significant differences emerged between the studied fermenters with regards to the hardness of the product in the mouth. This is a positive outcome given that olives processed under controlled temperatures had a higher fermentation rate and, therefore, may be ready for the market more quickly than control olives. A greater microbial load following examined treatments did not generate any defects or damaged olive texture. No undesirable softening of the olives was reported.

Sensory acidity was rated between 4 and 5.1 points for all fermenters. This parameter was strictly standardized in all experiments.

No sensory defects were registered for any of the samples. This shows that no abnormal fermentations occurred under the conditions chosen for the three experiments (Figure 3). This is crucial because it demonstrates that controlled thermal treatments are able to ensure the production of a safe and excellent product. Controlled thermal treatments also allowed for efficient microbiological control.

## 4. Conclusions

In this work, controlling the temperature of the brine improves the fermentation process and enhances some of the parameters related to table olive quality. In this sense, brine temperature influences the final phenolic fingerprint of table olives of the Manzanilla de Sevilla and Manzanilla Cacereña variety. In addition, the present study shows that Sevillian-style production processes can be applied to both Manzanilla de Sevilla and Manzanilla Cacereña green table olive varieties with successful results. In addition, in the sensory analysis, samples fermented without temperature controls showed lower scores.

Heat treatment combined with temperature monitoring during the fermentation of table olives constitutes an approach to enhance the quality of Spanish-style table olives. Important improvements of table olive quality can be achieved by maintaining fermenters at a constant temperature, especially during the winter months. This shortens the fermentation time, especially in regions where the fermentation process can become stuck due to adverse temperature conditions, leading to a low-quality product. The use of a heat exchanger with fermentation temperature monitoring is easy to implement. This could help to reduce the time needed to market the product, which can be shortened by at least 2–3 weeks in some cases. The disadvantage of this methodology is that it requires additional energy expenditure, an aspect that requires assessment by the industry.

Brine temperature influences the final phenolic fingerprint of Manzanilla de Sevilla and Manzanilla Cacereña variety table olives. In addition, the present study shows that Sevillian-style production processes can be applied to both Manzanilla de Sevilla and Manzanilla Cacereña green table olive varieties with outstanding results. This innovative industrial-scale approach represents an opportunity for the sector in Extremadura because it will be useful to increase its standing in the global market more quickly. This will enable the region to obtain an economic benefit with only a small initial outlay. However, further studies are needed to monitor the growing conditions of the olive tree orchards and examine other representative variables which may affect the production process.

## Figures and Tables

**Table 1:** Viable counts pertaining to total mesophiles, yeasts and mold, and lactic acid bacteria (LAB). Outcomes reported as mean ± SD (log_10_ cfu·g^−1^). Different superscript letters within the same column indicate statistically significant differences (Duncan’s test, *p* < 0.05) between thermal treatments. n.s.: non-significant differences.

**(A) Experiment 1**				
**Fermentation Day**	**Heat Treatment**	**LAB**	**Yeasts and Mold**	**Mesophile**
5	Control	1.24 ± 0.21 ^n.s.^	1.20 ± 0.25 ^n.s.^	1.42 ± 0.33 ^n.s.^
	Sheltered	1.29 ± 0.86	1.53 ± 0.32	1.22 ± 0.23
20	Control	2.25 ± 0.77 ^a^	3.45 ± 0.24 ^n.s.^	2.52 ± 0.28 ^n.s.^
	Sheltered	3.37 ± 0.05 ^b^	3.38 ± 0.49	3.03 ± 0.11
60	Control	3.99 ± 0.14 ^a^	3.54 ± 0.03 ^n.s.^	3.04 ± 0.19 ^a^
	Sheltered	5.33 ± 0.14 ^b^	3.11 ± 0.12	3.79 ± 0.13 ^b^
100	Control	3.85 ± 0.24 ^a^	4.07 ± 0.54 ^b^	3.62 ± 0.11 ^a^
	Sheltered	5.28 ± 0.12 ^b^	3.07 ± 0.54 ^a^	4.29 ± 0.14 ^b^
140	Control	3.12 ± 0.12 ^a^	4.79 ± 0.13 ^b^	4.55 ± 0.12 ^a^
	Sheltered	5.01 ± 0.22 ^b^	4.07 ± 0.21 ^a^	5.42 ± 0.22 ^b^
160	Control	2.80 ± 0.11 ^a^	5.01 ± 0.11 ^b^	5.05 ± 0.11 ^a^
	Sheltered	4.51 ± 0.11 ^b^	4.02 ± 0.12 ^a^	6.65 ± 0.15 ^b^
**(B) Experiment 2**				
**Fermentation Day**	**Heat Treatment**	**LAB**	**Yeasts and Mold**	**Mesophile**
5	Control	1.11 ± 0.11 ^a^	1.23 ± 0.13 ^a^	1.31 ± 0.23 ^n.s.^
	20	2.12 ± 0.13 ^b^	1.61 ± 0.11 ^c^	1.21 ± 0.10
	22	2.11 ± 0.12 ^b^	1.32 ± 0.14 ^b^	1.22 ± 0.14
	24	2.23 ± 0.13 b	1.50 ± 0.14 ^b^	1.32 ± 0.13 ^b^
20	Control	3.15 ± 0.24 ^a^	4.22 ± 0.22 ^a^	3.22 ± 0.23 ^a^
	20	5.82 ± 0.12 ^b^	4.01 ± 0.24 ^a^	3.76 ± 0.11 ^b^
	22	6.05 ± 0.22 ^c^	4.42 ± 0.15 ^b^	3.83 ± 0.12 ^b^
	24	6.33 ± 0.13 ^c^	4.53 ± 0.15 ^b^	3.81 ± 0.22 ^b^
60	Control	3.09 ± 0.11 ^a^	3.10 ± 0.10 ^a^	3.21 ± 0.11 ^a^
	20	5.31 ± 0.13 ^b^	4.11 ± 0.11 ^b^	4.02 ± 0.14 ^b^
	22	6.15 ± 0.21 ^c^	4.31 ± 0.14 ^c^	4.23 ± 0.14 ^b^
	24	6.54 ± 0.22 ^d^	4.42 ± 0.14 ^c^	4.41 ± 0.21 ^c^
100	Control	3.42 ± 0.21 ^a^	4.12 ± 0.23 ^ns^	3.42 ± 0.13 ^a^
	20	5.51 ± 0.22 ^b^	4.22 ± 0.21	4.32 ± 0.13 ^b^
	22	6.15 ± 0.31 ^c^	4.42 ± 0.13	4.11 ± 0.11 ^b^
	24	6.51 ± 0.32 ^d^	4.53 ± 0.32	4.32 ± 0.23 ^b^
140	Control	3.02 ± 0.22 ^a^	4.12 ± 0.12 ^a^	4.35 ± 0.12 ^a^
	20	6.4 ± 0.21 ^c^	4.5 ± 0.11 ^b^	5.21 ± 0.21 ^b^
	22	6.23 ± 0.11 ^b^	4.64 ± 0.12 ^c^	5.12 ± 0.21 ^b^
	24	6.44 ± 0.21 ^c^	4.72 ± 0.21 ^c^	5.71 ± 0.22 ^c^
160	Control	2.2 ± 0.1 ^a^	3.4 ± 0.1 ^a^	5.3 ± 0.1 ^a^
	20	4.3 ± 0.1 ^b^	4.2 ± 0.1 ^b^	6.3 ± 0.2 ^b^
	22	4.3 ± 0.1 ^b^	4.7 ± 0.1 ^c^	6.6 ± 0.2 ^b^
	24	4.4 ± 0.1 ^b^	4.2 ± 0.1 ^b^	6.7 ± 0.2 ^b^
**(C) Experiment 3**				
**Fermentation Day**	**Heat Treatment**	**LAB**	**Yeasts and Mold**	**Mesophile**
5	Control	1.52 ± 0.21 ^n.s.^	1.35 ± 0.23 ^n.s.^	1.45 ± 0.14 ^n.s.^
	Buried	2.02 ± 0.23	1.07 ± 0.24	1.54 ± 0.53
	Isolated	1.63 ± 0.22	1.33 ± 0.41	1.73 ± 0.42
20	Control	3.32 ± 0.12 ^n.s.^	4.11 ± 0.31 ^n.s.^	3.23 ± 0.22 ^n.s.^
	Buried	3.22 ± 0.24	3.01 ± 0.42	3.01 ± 0.58
	Isolated	3.31 ± 0.32	3.06 ± 0.32	4.02 ± 0.31
60	Control	3.73 ± 0.10 ^a^	3.11 ± 0.23 ^a^	3.33 ± 0.23 ^a^
	Buried	3.84 ± 0.22 ^b^	3.91 ± 0.22 ^b^	4.41 ± 0.11 ^b^
	Isolated	4.92 ± 0.22 ^c^	4.01 ± 0.22 ^c^	4.23 ± 0.24 ^b^
100	Control	4.23 ± 0.22 a	4.53 ± 0.12 ^n.s.^	4.31 ± 0.12 ^n.s.^
	Buried	5.02 ± 0.12 ^b^	4.21 ± 0.11	4.22 ± 0.21
	Isolated	6.01 ± 0.12 ^c^	4.06 ± 0.31	4.11 ± 0.12
140	Control	3.56 ± 0.21 ^a^	4.02 ± 0.11 ^a^	4.15 ± 0.13 ^n.s.^
	Buried	5.02 ± 0.11 ^b^	4.51 ± 0.21 ^c^	4.03 ± 0.11
	Isolated	5.93 ± 0.24 ^c^	4.32 ± 0.21 ^b^	4.13 ± 0.44
160	Control	2.31 ± 0.11 ^a^	3.01 ± 0.11 ^a^	5.01 ± 0.23 ^a^
	Buried	3.82 ± 0.12 ^c^	3.72 ± 0.12 ^b^	6.02 ± 0.22 ^b^
	Isolated	3.53 ± 0.13 ^b^	3.61 ± 0.12 ^b^	6.12 ± 0.11 ^b^

**Table 2:** Phenolic concentration of table olives (mg/kg) at the end of the fermentation process. Results are expressed as mean ± SD of the three sample replicates. Different superscript letters within the same columns indicate statistically significant differences (Duncan´s Test, *p* < 0.05) between thermal treatments. PB1 and PB2: procyanidin B1 and B2.

**(A) Experiment 1**						
**Heat Treatment**	**Hydroxytyrosol**	**Tyrosol**	**Oleuropein**	**Apigenin**	**Luteolin**	**Luteolin-7-O-Glucoside**
**Control**	551 ± 55 ^b^	126 ± 16 ^b^	108 ± 17 ^n.s.^	1.3 ± 0.4 ^n.s.^	8.4 ± 3.1 ^a^	2.6 ± 0.6 ^n.s.^
**Sheltered**	484 ± 84 ^a^	104 ± 14 ^a^	99 ± 19	1.3 ± 1.1	13.9 ± 3.8 ^b^	2.3 ± 0.7
	**Vanillin**	**Vanillic Acid**	**PB1**	**PB2**	**Epicatechin**	**Catechin**
**Control**	2.8 ± 0.8 ^n.s.^	4.1 ± 0.6 ^n.s.^	13.1 ± 3.7 ^n.s.^	2.4 ± 0.4 ^n.s.^	6.7 ± 0.7 ^b^	6.6 ± 1.5 ^n.s.^
**Sheltered**	2.4 ± 0.7	3.4 ± 2.5	14.8 ± 6.3	2.1 ± 0.6	4.6 ± 1.9 ^a^	7.7 ± 2.7
**(B) Experiment 2**						
**Heat treatment**	**Hydroxytyrosol**	**Tyrosol**	**Oleuropein**	**Apigenin**	**Luteolin**	**Verbascoside**
Control	1026 ± 50 ^n.s.^	198 ± 26 ^c^	124 ± 8 ^c^	0.6 ± 0.1 ^b^	4.0 ± 0.2 ^a^	1.7 ± 0.8 ^b^
20	963 ± 100	162 ± 22 ^b^	100 ± 6 ^a,b^	0.4 ± 0.1 ^a^	4.2 ± 0.2 ^b^	0.8 ± 0.4 ^a^
22	963 ± 82	144 ± 16 ^a^	109 ± 7 ^b^	0.8 ± 0.1 ^c^	5.7 ± 0.2 ^c^	0.7 ± 0.3 ^a^
24	929 ± 82	148 ± 32 ^a^	88 ± 8 ^a^	0.4 ± 0.1 ^a^	4.4 ± 0.2 ^b^	1.0 ± 0.1 ^a^
	**Vanillin**	**Vanillic Acid**	**PB1**	**PB2**	**Catechin**	**Epicatechin**
Control	8.2 ± 1.1 ^n.s.^	3.3 ± 0.2 ^n.s.^	15 ± 2 ^b^	2.8 ± 0.4 ^b^	7.3 ± 0.5 ^n.s.^	6.0 ± 0.8 ^n.s.^
20	8.3 ± 0.1	3.5 ± 0.4	15 ± 1 ^b^	2.2 ± 0.3 ^a^	8.0 ± 0.3	5.9 ± 0.5
22	10.9 ± 0.2	4.3 ± 0.3	15 ± 1 ^b^	2.7 ± 0.2 ^a,b^	7.9 ± 0.4	6.6 ± 0.7
24	11.6 ± 0.5	4.0 ± 0.3	10 ± 1 ^a^	2.6 ± 0.2 ^a,b^	7.9 ± 0.5	6.1 ± 0.3
**(C) Experiment 3**						
**Heat Treatment**	**Hydroxytyrosol**	**Tyrosol**	**Oleuropein**	**Apigenin**	**Luteolin**	***o*-Vanillin**
Buried	915 ± 245 ^n.s.^	153 ± 39 ^n.s.^	142 ± 39 ^a,b^	n.q.	4.7 ± 1.9 ^n.s.^	14.1 ± 3.6 ^b^
Older	859 ± 175	141 ± 28	115 ± 33 ^a^	n.q.	3.7 ± 0.7	9.3 ± 6.6 ^a^
Isolated	934 ± 175	151 ± 32	159 ± 32 ^b^	0.60 ± 0.27	5.1 ± 1.0	16.0 ± 5.3 ^b^
	**Vanillin**	**Vanillic Acid**	**PB1**	**PB2**	**Epicatechin**	**Catechin**
Buried	5.0 ± 1.5 ^a^	4.2 ± 1.5 ^n.s.^	17.1 ± 5.3 ^n.s.^	2.09 ± 0.27 ^b^	4.8 ± 1.4 ^n.s.^	7.9 ± 2.1 ^n.s.^
Older	4.7 ± 1.3 ^a^	3.5 ± 1.4	14.4 ± 5.2	1.75 ± 0.13 ^a^	5.57 ± 0.93	6.6 ± 2.0
Isolated	6.8 ± 1.6 ^b^	4.3 ± 1.2	17.8 ± 5.6	2.22 ± 0.44 ^b^	5.01 ± 0.98	8.5 ± 2.9

n.s. non-significant. n.q. non-quantified.
